# Establishing and validating regulatory regions for variant annotation and expression analysis

**DOI:** 10.1186/s12864-016-2724-0

**Published:** 2016-06-23

**Authors:** Alexander Kaplun, Mathias Krull, Karthick Lakshman, Volker Matys, Birgit Lewicki, Jennifer D. Hogan

**Affiliations:** QIAGEN Bioinformatics, 35 Gatehouse Drive, Waltham, MA 02451 USA

**Keywords:** TRANSFAC, Annotation, Promoter, Transcription start site, Transcription factor binding, Regulatory variants

## Abstract

**Background:**

The regulatory effect of inherited or *de novo* genetic variants occurring in promoters as well as in transcribed or even coding gene regions is gaining greater recognition as a contributing factor to disease processes in addition to mutations affecting protein functionality. Thousands of such regulatory mutations are already recorded in HGMD, OMIM, ClinVar and other databases containing published disease causing and associated mutations. It is therefore important to properly annotate genetic variants occurring in experimentally verified and predicted transcription factor binding sites (TFBS) that could thus influence the factor binding event. Selection of the promoter sequence used is an important factor in the analysis as it directly influences the composition of the sequence available for transcription factor binding analysis.

**Results:**

In this study we first establish genomic regions likely to be involved in regulation of gene expression. TRANSFAC uses a method of virtual transcription start sites (vTSS) calculation to define the best supported promoter for a gene. We have performed a comparison of the virtually calculated promoters between the best supported and secondary promoters in hg19 and hg38 reference genomes to test and validate the approach. Next we create and utilize a workflow for systematic analysis of casual disease associated variants in TFBS using Genome Trax and TRANSFAC databases. A total of 841 and 736 experimentally verified TFBSs within best supported promoters were mapped over HGMD and ClinVar mutation sites respectively. Tens of thousands of predicted ChIP-Seq derived TFBSs were mapped over mutations as well. We have further analyzed some of these mutations for potential gain or loss in transcription factor binding.

**Conclusions:**

We have confirmed the validity of TRANSFAC’s approach to define the best supported promoters and established a workflow of their use in annotation of regulatory genetic variants.

## Background

The paradigm that meaningful alterations in DNA sequence have to be in the coding regions of genes and must lead to significant changes in protein structure and functionality [1] has been long denounced with discovery of ever growing cohort of examples of striking effect of genetic variants in promoters or of synonymous changes in translated areas of exons [2–4]. Appropriate annotation of such regulatory variants represents one of the biggest challenges in analysis of Next Generation Sequencing (NGS) data. In general, annotation relies on databases consolidating published reports of disease causing germline (HGMD [5], OMIM [6], ClinVar [7]) and somatic (COSMIC [8], TCGA [9]) mutations or pharmacogenomic variants (PharmGKB [10], PGMD [11]) which include multiple regulatory mutations.

In this context, experimentally verified transcription factor binding sites (TFBSs), which overlap with variants in non-coding regions are of particular importance. TRANSFAC [12], the most complete manually curated database in the field of gene regulation, includes information on tens of thousands of TFBSs currently reported in peer-reviewed literature. Unfortunately, currently available data is far from being comprehensive. While for a handful of well-studied genes such as TP53 or BRCA1, which attract significant attention of scientific community, fifty or more TFBSs may have been reported, less studied genes usually have very few experimentally verified TFBS or even none at all. Thus using only reported binding sites as means to predict or explain the relevance of a genetic variant in NGS annotation will produce incomplete or even misleading results, which may need to be complemented by predictions. Needless to say that most of the traditional predicting algorithms routinely used to estimate impact of mutations as SIFT [13], Polyphen [14] etc., cannot be used in such cases since they are based on estimation of changes in protein structure or conservation of protein sequence.

TRANSFAC implements Match algorithm [15] for prediction of potential TFBSs through comparison of an input DNA sequence with a library of Positional Weight Matrices (PWMs) as consensus derived from experimentally verified TFBSs. While predictions made by Match are often remarkably accurate, the algorithm is based solely on DNA sequence and is insensitive to location of predicted sites relative to promoters or Transcription Start Sites (TSSs). Thus selection of the promoter sequence used is an important factor in the analysis as it directly influences the composition of the sequence available for transcription factor binding. Traditionally promoters are defined as intervals relative to TSSs, however number and position of reported TSSs varies from gene to gene. TSSs are derived from experimental mRNA sequences and can be very close to each other or thousands on nucleotides apart. Using Match analysis to estimate regulatory effect of genetic variants near all experimentally verified TSSs would be the most comprehensive approach, however number of known transcripts for typical gene can exceed 100 with tendency to grow over time and their TSSs may span over tens of thousands base pairs. Many variants, particularly in cases of whole genome or whole exome sequences will map over these regions, leading to unacceptable level of false positive hits and masking variants actually affecting gene regulation. For effective filtering of NGS data it is thus necessary to determine which regions are most likely to play regulatory role in majority of cases.

In this study we first establish genomic regions likely to be involved in regulation of gene expression. TRANSFAC uses a method of virtual TSS calculation to define the best supported promoter for a gene. We perform a comparison of the virtual promoters between the best supported and secondary promoters in hg19, as well as in hg38 reference genomes to test and validate the approach. Next we create and utilize a workflow for systematic analysis of casual disease associated variants in TFBS using Genome Trax [16] and TRANSFAC databases. A total of 841 and 736 experimentally verified TFBSs within best supported promoters were mapped over HGMD and ClinVar mutation sites respectively. Tens of thousands of predicted ChIP-Seq derived TFBSs were mapped over mutations as well. We have further analyzed some of these mutations for potential gain or loss in transcription factor binding.

## Results and discussion

### Classification of promoters

When analyzing promoter properties such as the pattern of distribution of transcription factor binding sites and other features we considered three distinct groups of promoters: single promoters, best supported promoters, and secondary promoters. Single promoters are designated as such because their vTSS was the only one identified for the associated gene. As described in the Methods section, best supported promoters are those promoters whose vTSS is the best scoring for a gene with multiple vTSSs while secondary promoters are all other promoters that are not either a single promoter or a best supported promoter.

### Human FGFR1 as an example case

Working with TRANSFAC version 2014.4, which is based upon EnsEMBL [17] version 76 using reference genome hg38, we classified each human promoter. Taking FGFR1, a fibroblast growth factor receptor and protein tyrosine kinase that plays a role in cell proliferation and skeletal development, as an example, two vTSSs were identified (Table 1).

**Table 1:** vTSSs of FGFR1

Chromosome	Position	Clustering score	Percent	Best supported
8	38468271	95	46.3 %	Yes
8	38457480	25	12.2 %	No

The vTSS at position 38468271 was identified as the best supported based on the clustering score of 95. The remaining vTSSs defines a secondary promoter.

**Fig. 1:**
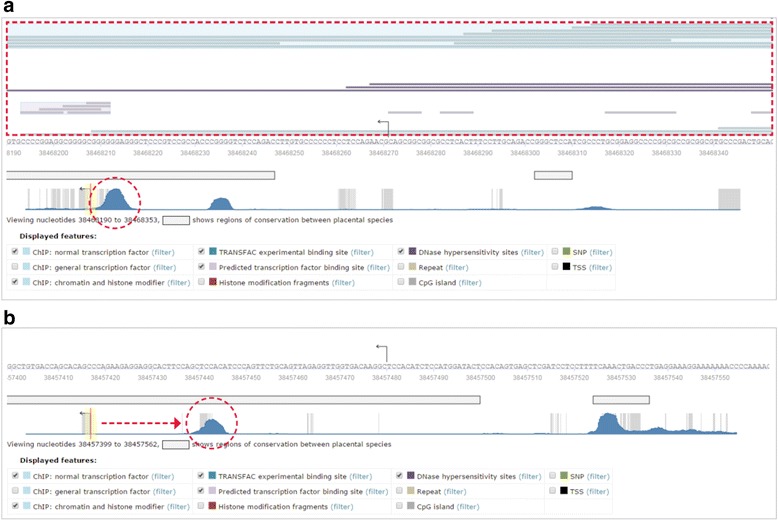
Screenshot of a part of promoter report showing best supported (**a**) and secondary (**b**) promoters of FGFR1 generated via TRANSFAC Professional online interface. Overlapped features include ChIP-on-chip/-seq fragments (identifies DNA fragments shown in vivo to be bound by a transcription factor, based on ChIP-seq or related experiments), Histone modifications (identifies DNA fragments shown in vivo to be bound by histones with a particular modification, based on ChIP-seq or related experiments), DNase hypersensitivity sites derived from ENCODE, TSSs (identifies transcription start sites from Ensembl), TRANSFAC experimental and predicted sites and more. Most of these features can be filtered by cell type used in the experiment, transcription factor, position etc

Looking at the graphical display of the best supported promoter (Fig. 1a) we can see by the gray bar underneath the zoomed in nucleotide sequence flanking the vTSS, determined by phastcons score, that this region of the genome is well conserved when compared to the mouse genome. We can also see that numerous mapped features including ChIP fragments and predicted transcription factor binding sites within DNase I hypersensitivity sites are present at the vTSS shown by the blue and purple bars above the sequence, and become even more concentrated just upstream of the vTSS shown by the blue peak in the zoomed out full promoter view shown directly above the legend.

In contrast, looking at the sequence that flanks the vTSS of the secondary promoter located at position 38457480, we see a similar level of conservation but no mapped features surrounding or lying immediately upstream of the vTSS (Fig. 1b).

### Promoter characterization across the genome

**Fig. 2:**
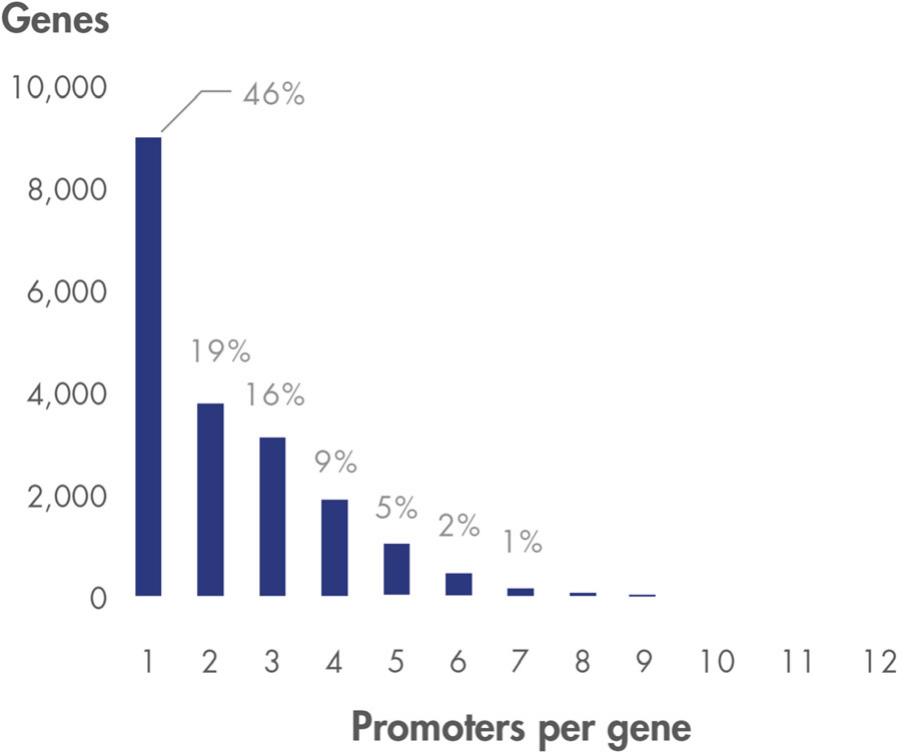
Distribution of number of promoters of protein-encoding genes determined by vTSS algorithm described in Methods section

While these examples provide a detailed view of two individual promoters, for a broader view of promoter distribution across the genome we looked at the number of promoters identified for each human gene. We found that the number of promoters per protein-encoding gene ranged from 1 to 12, with 46 % having a single promoter and 81 % having three promoters or fewer (Fig. 2).

As a way of assessing the quality of the single, best supported and secondary promoters we looked at the distribution of overlapping transcription factor binding sites. Two types of transcription factor binding sites were considered – TRANSFAC experimentally verified binding sites and TRANSFAC predicted binding sites within ChIP-Seq and DNase I hypersensitivity fragments. The experimentally verified binding sites are literature-curated transcription factor binding sites that have been individually studied and validated. Predicted binding sites are experimental binding sites which have been refined by prediction. ChIP-seq fragments are typically hundreds of nucleotides long. It is known which factor binds them, but not exactly where in the sequence the factor binds. The most conserved, relevant TRANSFAC PWMs for the factors are used for the analysis with the minFP matrix cut-off to minimize false positives, and the best scoring sites are calculated with the Match algorithm executed with an option to return one best hit in the whole sequence. By limiting the site prediction to a predefined transcription factor and a short ChIP-seq fragment, there is low risk of identifying false-positive binding sites in this process. The majority of the ChIP-Seq data are derived from ENCODE. This data is somewhat biased due to over-representation of a few commonly used cell lines.

Hypersensitivity to DNase correlates with the presence of regulatory elements in the neighborhood of genes. DNase sensitive fragments are typically hundreds of nucleotides long. It is not known which factors bind them, or where. 142 ENCODE data sets [18] based on different cell lines were collected and potential transcription factor binding sites on the DNase fragment sequences were identified by running the Match algorithm using a non-redundant set of 148 high quality matrices from vertebrates with the minFP matrix cut-off to minimize false positives and an option to return the one best hit for the matrix in the whole sequence, to generate maximally one high scoring site, for each sequence and matrix.

**Fig. 3:**
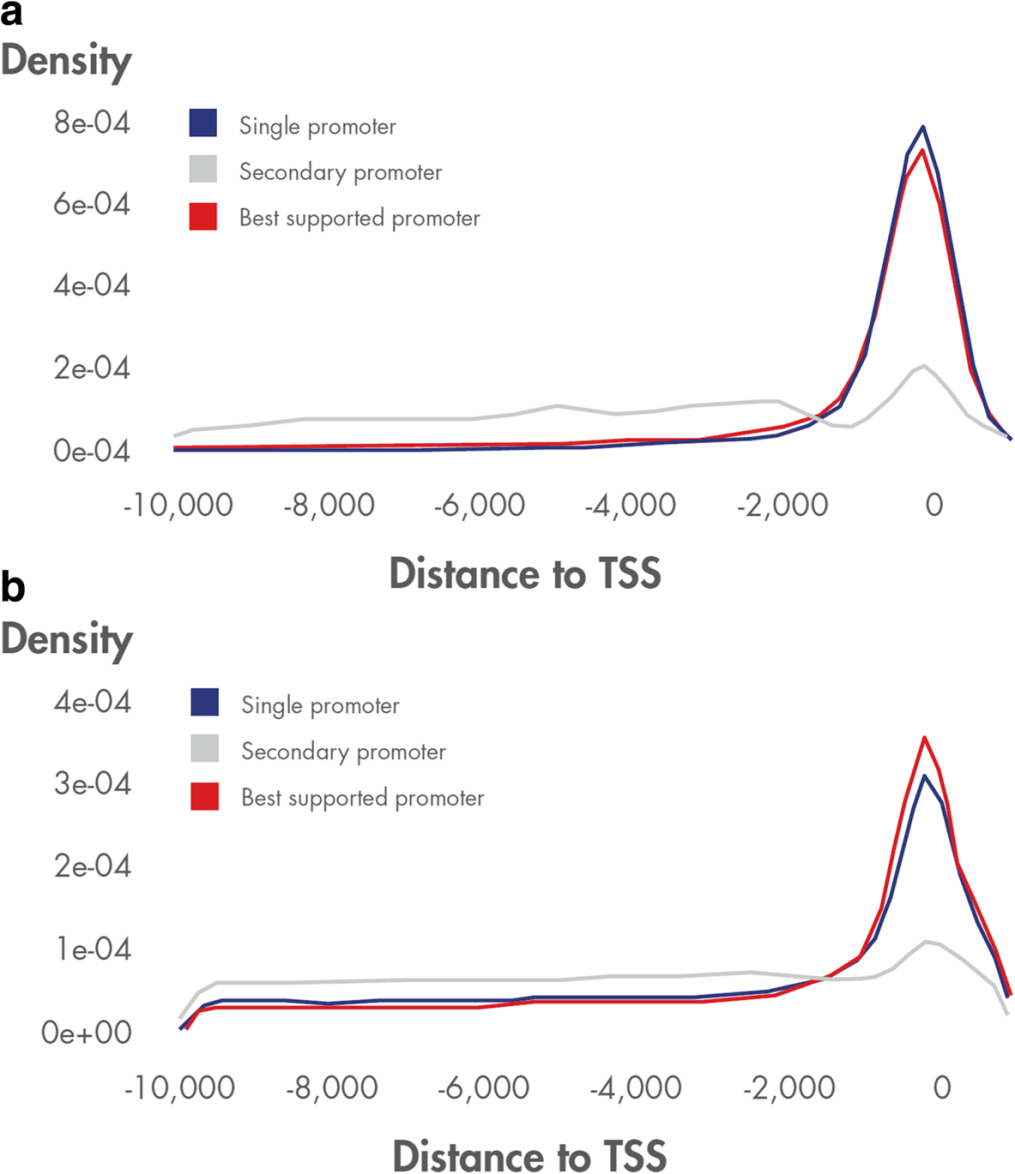
Experimental (**a**) and predicted (**b**) TFBS distance to vTSS. The graphs are plots of kernel density estimations and the definite integral over its support set equals to 1

Distribution of experimental binding sites clearly clusters around the vTSS for single and best supported promoters, whereas distribution around secondary promoters looks to be less structured and approaching a random distribution of sites (Fig. 3a). Predicted binding sites show a similar pattern of distribution (Fig. 3b) with somewhat higher background density. The peak around the vTSS is less pronounced for the predicted sites, due to a higher background of false positive or non-functional site predictions. The background noise is evenly distributed in the graph due to the high number of PWMs involved as well as to the smoothing effect of the density function.

### Comparison of promoters calculated for hg38 versus hg19 reference genome

Interested in the relative stability of promoter assignments when a different reference genome is used as input, we compared the distribution of promoters between TRANSFAC version 2014.4, which is based upon EnsEMBL version 76 using reference genome hg38, and TRANSFAC version 2014.3, which is based upon EnsEMBL version 75 using reference genome hg19.

**Fig. 4:**
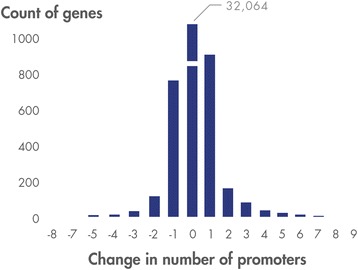
Change in promoter count between hg19 and hg38 genome builds

We first looked at the number of genes for which the count of promoters changed between the hg19 and hg38 reference genomes. Of the 36,462 protein- and RNA-encoding genes identified in the hg19 genome, 32,064 or 88 % showed no change in the number of promoters. That number increases to 33,737 or 93 % when a change of +/− 1 promoter is allowed (Fig. 4).

Two thousand two hundred twenty-eight genes (6 %) are excluded from the statistics due to the EnsEMBL ID having changed, mostly due to deprecated IDs or gene clusters.

In addition to looking at how the absolute count of promoters changed across the set of genes we also looked at how the promoters themselves changed by comparing the positions of the vTSSs between the hg19 and hg38 reference genomes for all promoters as well as the best supported promoters. From a total of 71,118 promoters identified in the hg19 reference genome 77–82 % remain unchanged, a range that increased to 83–86 % when a shift of 10 or more fewer nucleotides was allowed (Table 2).

**Table 2:** Change in positions of vTSSs between genome builds hg19 and hg38

	All promoters	% All promoters	Best supported promoters	% Best supported promoters
Unchanged	58,077	82 %	35,343	77 %
Shifted 1–10 nt	3,179	4 %	2,693	6 %
Shifted 11–100 nt	4,184	6 %	3,465	7 %
Shifted 101–1,000 nt	1,455	2 %	952	2 %
Total	71,118		45,608	

The remaining 6 % of all promoters and 8 % of best supported promoters either shifted more or dropped out due to the EnsEMBL ID having changed, mostly due to deprecated IDs or gene clusters. We believe that the larger shift that is observed for best supported promoters relative to all promoters may be due to a shift in relative scores that resulted in switching of the best supported promoter, but further investigation will be required to test this hypothesis.

### Profiling regulatory variations in transcription factor-binding sites associated with disease

We have selected three representative disease associated mutations out of 841 overlapping with TBFSs (see Methods) and estimated potential change in TF binding caused by these mutations. These cases were selected due to multiple reported mutations within affected TFBS and published experimental confirmation of their effect on gene regulation.

Four reported point mutations causing Charcot-Marie-Tooth disease [19] are located within Sox10 binding site in GJB1 promoter are shown in Table 3. Sox10 is known to strongly activate expression of GJB1 in vitro by direct binding to its promoter [20]. Some, but not all of these mutations were reported to affect this binding [21, 22].

**Table 3:** Mutations in Sox10 binding site in GJB1 promoter causing Charcot-Marie-Tooth disease

Chromosome	Sox10 site start	Sox10 site end	Variant coordinate	Variation
X	70443016	70443033	70443018	C > G
X	70443016	70443033	70443029	T > G
X	70443016	70443033	70443029	T > C
X	70443016	70443033	70443031	G > C

**Fig. 5:**
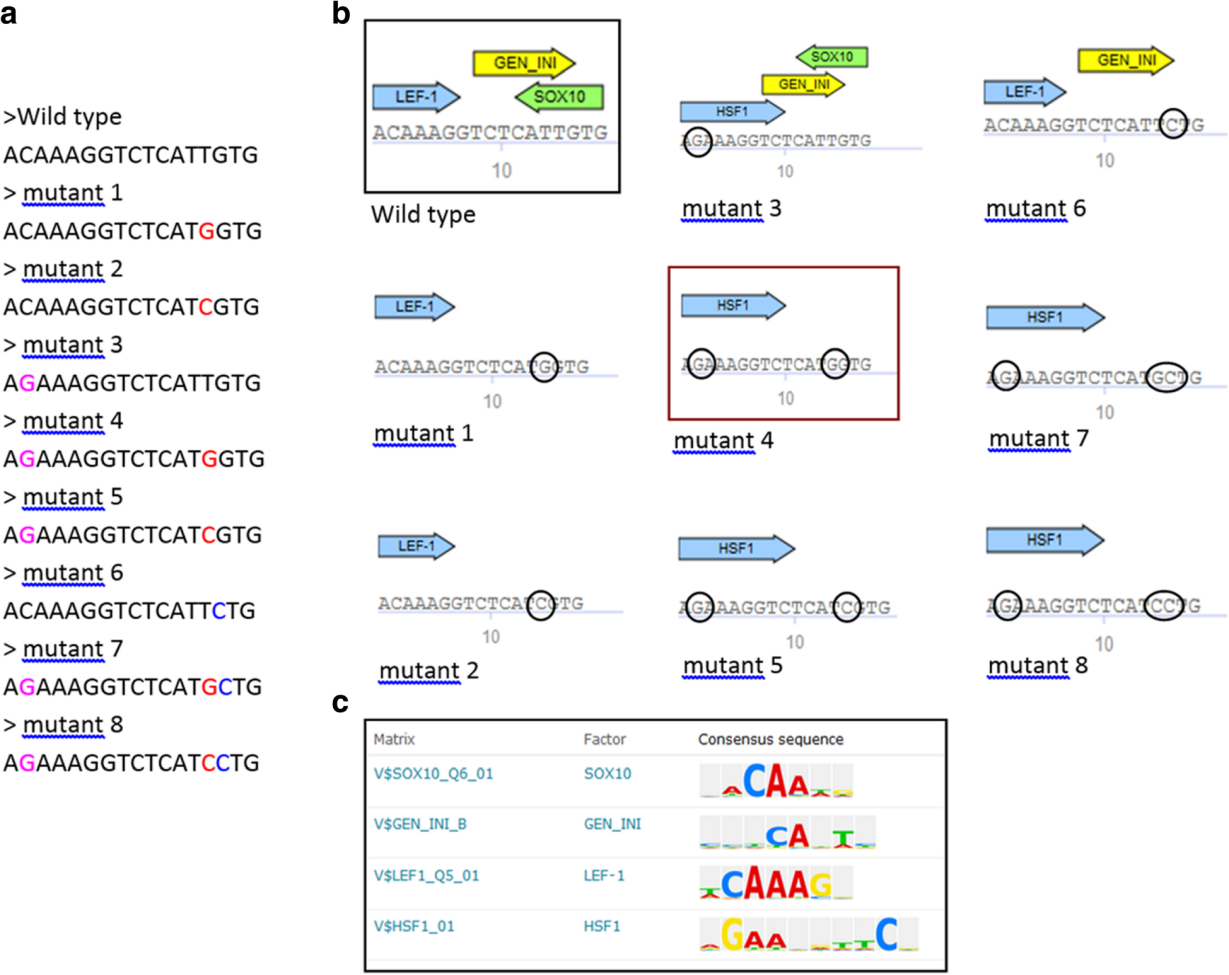
Match analysis of Sox10 binding site mutations causing Charcot-Marie-Tooth disease. **a** Sox10 binding sequence with analyzed disease causing variations highlighted in different colors. **b** Match output with TFBS, mutation sites are circled. **c** PWMs predicted to bind within the analyzed sequence

To study the effect of these changes on TF binding, the sequence region of GJB1 promoter which is specific for Sox10 binding (chrX:70443016–70443033) was extracted from TRANSFAC and various combinations of TFBS sequences were created with variations as shown in Fig. 5a. These sequences were used as Match analysis input to investigate the loss/gain of Sox10 binding depending on the variations in the sequence, as well as to detect other TFBS potentially affected by these mutations. As shown in Fig. 5b, mutations at positions 14 and 15 are predicted to abolish Sox10 binding as has already been reported [20]. Interestingly, mutation at position 2 abolished predicted LEF-1 binding site and created new site for HSF1 binding (Fig. 5b, c).

**Fig. 6:**
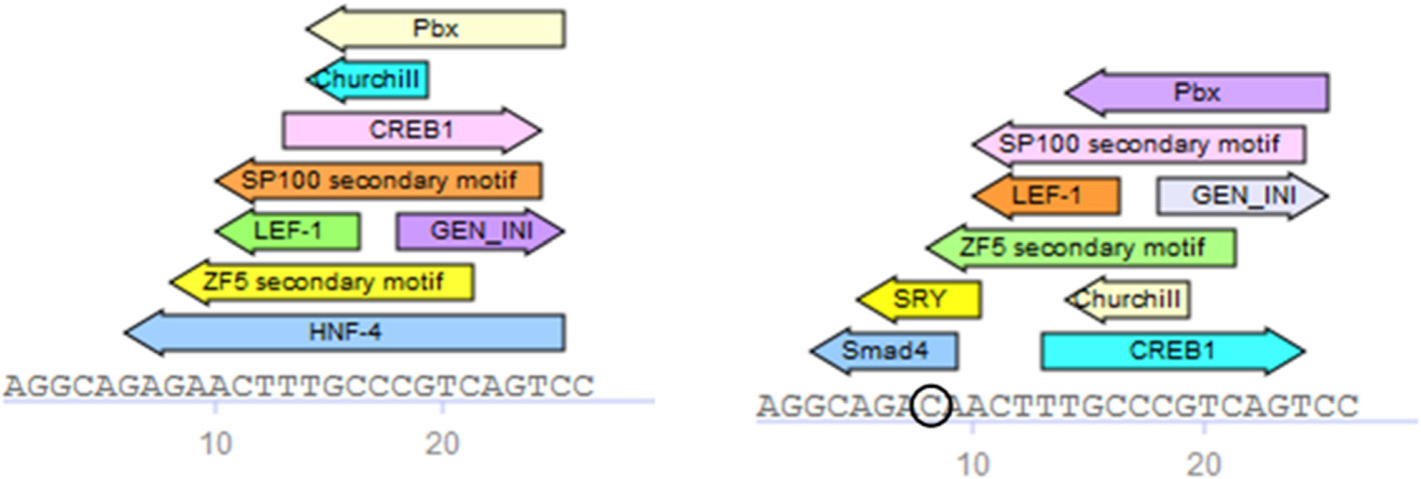
Match analysis of HNF4 binding region of F7 promoter with and without representative mutation causing Factor VII deficiency

Second example is multiple mutations within HNF-4 binding site in the promoter of F7 gene (Table 4). These mutations are reported to cause Factor VII deficiency, affecting HNF-4 regulation of F7 expression [22]. An SNV G > C at the position 8 of the binding site (at coordinate 113760091) not only abolishes HNF-4 binding as other mutations, but also introduces sites for Smad4 and SRY transcription factors as shown in Fig. 6.

**Table 4:** Mutations in HNF-4 binding site in F7 promoter causing Factor VII deficiency

Chromosome	HNF-4 site start	HNF-4 site end	Variant coordinate	Variation
13	113760083	113760109	113760091	G > C
13	113760083	113760109	113760094	C > T
13	113760083	113760109	113760095	T > G
13	113760083	113760109	113760096	T > G
13	113760083	113760109	113760097	T > G
13	113760083	113760109	113760099	C > T
13	113760083	113760109	113760101	C > T

**Fig. 7:**
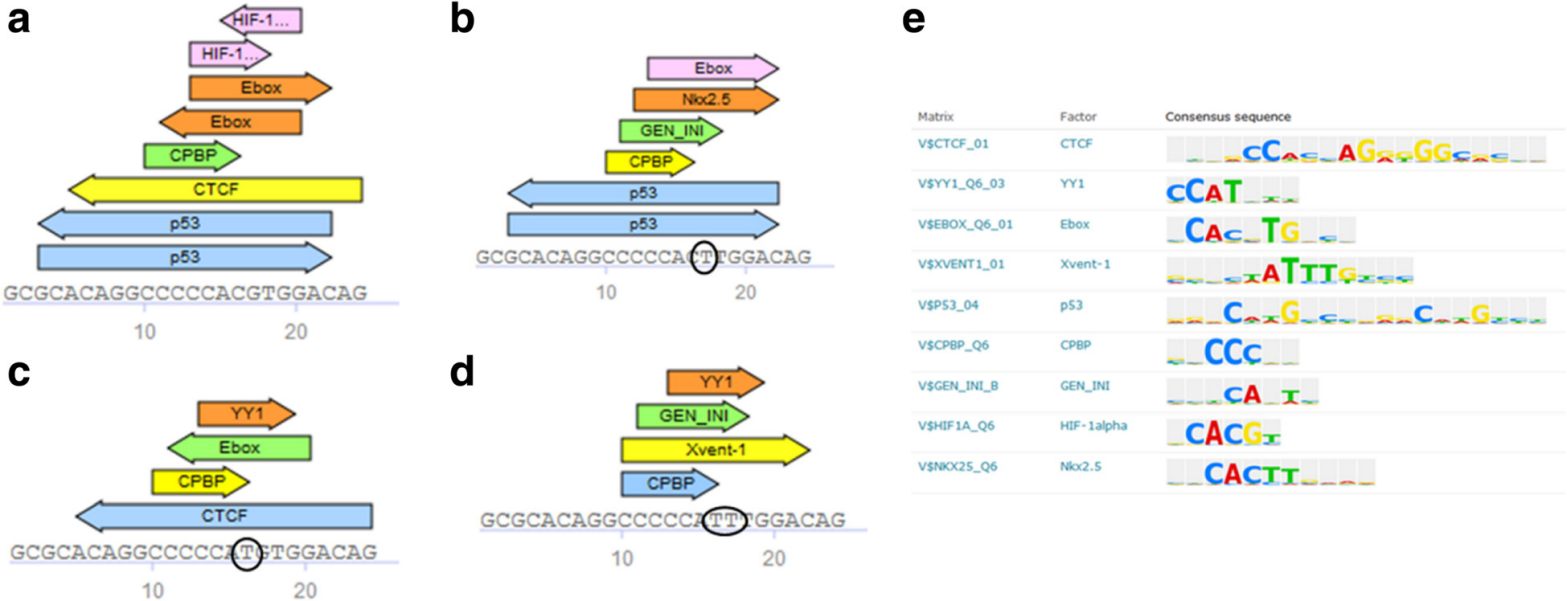
Analysis of HIF1-alpha binding site in ENG gene promoter affected by reported disease causing mutations associated with hereditary hemorrhagic telangiectasia (**a**-**d**) and PWMs predicted to bind within the analyzed sequence (**e**)

Another example is mutations in HIF1-alpha binding region in ENG promoter associated with hereditary hemorrhagic telangiectasia [23]. Match analysis suggests that binding is lost by G > T mutation at position 17 in the sequence while mutation at position 16 also abolishes p53 binding site (Fig. 7).

Basing on these examples one can extrapolate that results of Match analysis of disease causing mutations affecting regulation of gene expression in many cases are consistent with experimental data where available. Thus using Match predictions in analysis of variants located within regions of best supported promoters with highest frequency of TFBS and for which experimental data is limited could be very valuable both for diagnostics and for research of disease mechanisms.

## Conclusion

TRANSFAC’s approach to promoter selection, which is based on virtual TSS calculation and relative evidence levels, produces a set of promoters that are classified as single, best supported and secondary promoters. A specific comparison of the best supported and secondary promoter for human FGFR1 demonstrates a level of sequence conservation and clustering of characterized transcription factor binding sites near the vTSS for the best supported promoter that would be expected of a bona fide promoter, while clustering was less apparent for the weaker secondary promoter. When extended to the entire set of human promoters the clear clustering of characterized transcription factor binding sites held up, whereas the distribution around secondary promoters was confirmed to be less structured and suggestive of a random distribution.

One of major limitations of this approach is the fact that alternative promoters and isoforms may be specific of particular tissue, cell cycle phase or environmental conditions. Unfortunately, the majority of the available ChIP-seq data are from a relatively small range of generally used cell lines (an exception is CTCF which is involved in chromatin modification), thus the used ChIP-seq data do not represent all diversity of in vivo gene regulation. For the DNase hypersensitivity sites the data are derived from a larger set of different cell lines and tissues, but may still not cover all of them. The experimental environments for the individual sites are usually more varied, however still may have a bias, as certain cell lines are used more frequently in the laboratory practice than others.

We understand that our approach for defining and validating the best supported promoters does not take tissue-specific use of alternative promoters into account. Thus, for individual genes the promoter actually used in a particular tissue may deviate from the “best supported” promoter. However our data indicate that in general the “best supported” promoters are supported by different lines of evidence and that they allow to increase efficiency of NGS data filtering as well as significance of results of comparative promoter studies based on gene-specific microarray experiments (FMatch result, data not shown). In cases where transcript-specific information is available, as RNA-seq, we propose to use the TSSs of the actual transcripts as reference points for analysis.

Prediction algorithms, such as Match analysis, of disease causing and disease associated mutations could be introduced in routine of NGS annotation process, particularly if the detected variants are located in best supported promoters and within a range of vTSS that contains majority of experimentally verified TFBS. Such approach could compensate for limited experimental findings suitable for direct annotation of regulatory effects, and complement the array of prediction tools used for estimation of effect of mutations on protein functionality.

## Methods

### Selection of genomic sequences

Genomic sequence assemblies created by the international sequencing consortia are extracted from the EnsEMBL database. Promoter sequences are extracted through the process of virtual transcription start site calculation for all EnsEMBL genes of type protein- or miRNA-encoding. Genes on mitochondria are excluded, due to their special modes of transcription.

### Calculation of Virtual Transcription Start Sites (vTSSs)

The calculation of ‘virtual TSSs’ as reference points for promoter extraction is based on a collection of TSSs for a given gene. TSSs are taken as the first nucleotide of the most 5' exon of an EnsEMBL mRNA model. As multiple mRNA models may exist for a given gene, and those models may have very different start sites, collected TSSs for a given gene are often widespread throughout the sequence instead of located in tight clusters of only a few dozen nucleotides in length.

In order to define a reasonable number of vTSSs for a given gene from this data collection, an algorithm was designed which applies a set of rules to the data collection in order to find ‘clusters’ of TSSs. A window of 3000 nucleotides in length is slid along the sequence fragment defined by the set of TSSs for a given gene. A ‘clustering score’ is calculated by summing up weighted contributions from each TSS in the window. Each TSS derived from an EnsEMBL mRNA model starts with a score of 5. The scores are then weighted by multiplying by a distance score: the central position is multiplied by 1, the outer positions are multiplied by 0, and all positions in between by a value taken from a cosine function, according to the distance from the center of the window. The peaks of the resulting clustering score are regarded as potential vTSSs.

### Promoter selection and extraction

The set of potential vTSSs for a given gene is analyzed further to determine which will be used for promoter selection and extraction. For genes meeting the minimum cut-off for cumulative vTSS score, all vTSSs with a score that represents 8 % or more of the total are selected to define a promoter. The vTSS with the greatest percentage score is selected and defines what TRANSFAC describes as the best supported promoter. All other vTSSs, if present, are selected and define what TRANSFAC describes as secondary promoters.

Promoter sequences are determined using the genomic coordinate of the vTSS as the starting point. The bounding genomic coordinates that lie 10,000 nucleotides upstream of the vTSS and 1,000 nucleotides downstream of the vTSS are calculated and used to extract the intervening sequence. The calculation of vTSSs and the subsequent data extraction are fully automated processes.

### Exceptions

For some genes only a handful of evidence points are available, thus resulting in multiple virtual TSSs, each consisting of only a few evidence points. For all genes with a sum of vTSS scores less than the minimum cut-off, the most 5' vTSS is selected as the sole vTSS for the gene. For cases where there are two equally prominent peaks, the most 5' of the two vTSSs is selected to define the best supported promoter and all others are selected to define secondary promoters.

### Profiling disease-associated variations in TFBSs within best supported promoters

We have analyzed disease causing and disease associated mutations overlapping with experimentally verified TFBS located within intervals of −500 to +100 bp relative to vTSSs of 19,398 best supported promoters of human protein coding genes using Genome Trax annotation database. A total of 841 HGMD and 736 ClinVar mutations occurring in TFBS have been detected. Using Match analysis we have then evaluated the impact of some of these mutations on gain or loss of transcription factor binding affinity. For the analysis we have used TRANSFAC Professional 2014.3 data and non-redundant set of 148 high quality PMWs from vertebrates (provided with TRANSFAC) with the minSUM matrix cut-off.

## Ethics approval and consent to participate

Not applicable.

## Consent for publication

Not applicable.

## Availability of data and material

The datasets used in the analyses are parts of Transfac Professional and Genome Trax databases available at http://www.biobase-international.com/.

## Abbreviations

NGS: next generation sequencing
PWM: positional weight matrix
TFBS: transcription factor binding site
vTSS: virtual transcription start site
